# Effectiveness of Antivirals Nirmatrelvir-Ritonavir and Molnupiravir in Viral Sepsis: Retrospective Cohort Study

**DOI:** 10.2196/72124

**Published:** 2025-09-18

**Authors:** Teddy Tai Loy Lee, Alex Chang-Hao Lyu, Ting Ting Jiang, Sunny Ching Long Chan, Crystal Ying Chan, Edmond Tsz Fung Yip, Luke Yik Fung Luk, Joshua Wing Kei Ho, Kevin Wang Leong So, Omar Wai Kiu Tsui, Man Lok Lam, Shi Yeow Lee, Tafu Yamamoto, Chak Kwan Tong, Man Sing Wong, Eliza Lai Yi Wong, Abraham Ka Chung Wai, Timothy Hudson Rainer

**Affiliations:** 1 Department of Emergency Medicine, The University of Hong Kong, G06, 5 Sassoon Road, Pokfulam, Hong Kong, China (Hong Kong), 852 94189632; 2 Centre for Health Systems & Policy Research, JC School of Public Care & Primary Care, Faculty of Medicine, The Chinese University of Hong Kong, Hong Kong, China (Hong Kong); 3 School of Biomedical Sciences, Li Ka Shing Faculty of Medicine, The University of Hong Kong, Hong Kong, China (Hong Kong); 4 Yan Chai Hospital, Hong Kong Hospital Authority, Hong Kong, China (Hong Kong); 5 The University of Hong Kong, Hong Kong, China (Hong Kong); 6 Department of Land Surveying and Geo-informatics, Faculty of Construction and Environment, The Hong Kong Polytechnic University, Hong Kong, China (Hong Kong)

**Keywords:** organ dysfunction, viral infection, sepsis, antivirals, COVID-19

## Abstract

**Background:**

Viral infections, including those leading to sepsis, are common but often overlooked in clinical practice, yet the treatment strategies for viral sepsis remain inadequately defined.

**Objective:**

This study aims to investigate the effectiveness of antivirals nirmatrelvir-ritonavir and molnupiravir in the treatment of culture-negative sepsis.

**Methods:**

This retrospective cohort study was conducted across public hospitals in Hong Kong. We included patients diagnosed with COVID-19 between February 22, 2022, and June 30, 2023, who had no secondary bacterial or fungal infections. Propensity score matching was used to assess the efficacy of the antivirals nirmatrelvir-ritonavir and molnupiravir in patient subgroups with or without organ dysfunction at hospital admission, including circulatory shock, respiratory failure, acute kidney injury, coagulopathy, acute liver impairment, a composite of all organ dysfunctions, or no organ dysfunction. Key outcomes were in-hospital mortality and length of stay, reported as hazard ratios (HR) and mean differences, respectively.

**Results:**

The study included 15,599 COVID-19 patients with a mean age of 75.1 (SD 15.9) years. Molnupiravir treatment was associated with a significantly lower risk of mortality in patients in both the presence of any organ dysfunction (HR 0.75, 95% CI 0.58 to 0.96) and without organ dysfunction (HR 0.29, 95% CI 0.15-0.56). Nirmatrelvir-ritonavir was associated with decreased mortality with respiratory failure (absolute risk difference: 9.5%, 95% CI 6.26-12.72) and without organ dysfunction (HR 0.17, 95% CI 0.05-0.56). Antivirals also reduced the length of hospital stay; nirmatrelvir-ritonavir reduced length of stay in respiratory failure by an average of 3.37 (95% CI 2.32-4.42) days, acute kidney injury by 7.25 (95% CI 2.97-11.52) days, and coagulopathy by 7.04 (95% CI 2.99-4.05) days. Molnupiravir reduced the length of stay in acute kidney injury by an average of 6.7 (95% CI 2.39-11.08) days and coagulopathy by 5.68 (95% CI 1.20-10.16) days.

**Conclusions:**

Antivirals reduced mortality among hospitalized COVID patients, with the greatest reduction observed in patients without organ dysfunction. Antivirals were also effective in reducing the length of hospital stay.

## Introduction

Sepsis, a syndrome characterized by organ dysfunction resulting from a dysregulated response to infection, poses a significant mortality risk [1]. While bacterial and fungal infections are well-recognized causes of sepsis, viral infections—especially in older people and immunocompromised individuals—are often overlooked despite their potential to cause severe illness [2].

Extensive literature has primarily focused on bacterial sepsis, with early antibiotic administration being the cornerstone of its treatment [3,4]. However, the emergence of COVID-19, caused by the SARS-CoV-2, has sparked renewed interest in the role of viral infections in sepsis. Despite ongoing vaccination efforts that have reduced the severity and mortality associated with COVID-19 [5], the disease continues to significantly impact vulnerable populations, particularly older people.

A meta-analysis revealed that the majority of COVID-19 patients admitted to the ICU met the Sepsis-3 criteria [6]. During the early phases of the pandemic, COVID-19 accounted for 1 in 6 sepsis cases [7]. COVID-19 is associated with a range of organ dysfunctions, including circulatory shock, respiratory failure, acute kidney injury, liver impairment, and coagulopathy [8]. Yet, the use of antivirals in patients across varying organ dysfunction presentations remains inadequately understood. On a related note, recent studies support nirmatrelvir-ritonavir use in other critical conditions, such as in patients with advanced kidney disease [9], in hematologic malignancies [10], and in immunosuppressed patients [11], where antivirals reduced length of stay, shortened time to symptom resolution, and reduced viral load.

Viral sepsis remains underdiagnosed due to its lack of specific clinical manifestations [12]. Early research indicates that viral sepsis often occurs without secondary bacterial and fungal infections; for instance, 76% of blood and sputum cultures from COVID-19 patients were negative for bacteria and fungi [13].

Currently, guidelines offer limited recommendations for the use of antivirals in patients with viral sepsis [14,15], highlighting the need for further research to establish effective treatment protocols. This study aims to evaluate the effectiveness of antiviral treatment in a cohort of COVID-19 patients who had negative blood culture results during their hospital stay, thereby contributing to the evidence base for managing viral sepsis.

## Methods

### Cohort Identification

All patients included in this study were identified using the Clinical Data Analysis and Reporting System (CDARS), a comprehensive territory-wide electronic health care database encompassing all public hospitals managed by the Hospital Authority, which serves a population of 7.1 million. CDARS has been reliably used for numerous large-scale population-based studies [16], including research on antiviral use for COVID-19 [17,18], where diagnoses are stored using *International Classification of Diseases, Ninth Revision, Clinical Modification* (*ICD-9-CM*) codes. Each patient’s data, including prescription, admission, and diagnosis, were linked using a unique anonymous identifier. Patients were potentially included in this study if they had related service records, such as admission and medication use within CDARS.

### Study Design

This retrospective cohort study used CDARS to investigate culture-negative viral infection among COVID-19 inpatients. The study compared the effect of antiviral treatments (nirmatrelvir-ritonavir or molnupiravir) versus no antiviral treatment (control) on 30-day in-hospital mortality and length of stay, across various conditions of organ dysfunction.

### Eligibility Criteria

The cohort selection is detailed in Figure S1 in Multimedia Appendix 1. Eligible patients were those aged 18 years and older with a diagnosis of COVID-19, identified using *ICD-9-CM* code 519.8(8). Patients admitted between February 22, 2022, and June 30, 2023, were included in the study. The baseline time was defined as the date of hospital admission. To meet the culture-negative criteria, patients had to have at least one blood culture record during admission. Within the first 21 days of admission, no organism should have been identified from the culture, or only “negative organisms” should have been considered irrelevant to the viral infection course, as defined in Table S1 in Multimedia Appendix 1. Only patients who were culture-negative were included. As the study focus is on sepsis, other cultures, such as sputum culture, were not used to fulfill the culture-negative criteria; moreover, viral co-infections were not excluded, as they are within the scope of the study.

### Organ Dysfunction

Patients were categorized into one of 6 organ dysfunction groups (circulatory shock, respiratory failure, acute kidney injury, acute liver impairment, coagulopathy, composite organ dysfunction) based on the presence of organ dysfunction within the first 3 days of admission, as defined in Table S2 in Multimedia Appendix 1. These groups use the same method used to identify organ dysfunction according to the Hospital Toolkit for Adult Sepsis Surveillance released by the US Center for Disease Control and Prevention [19]. Patients without any organ dysfunction during the entire follow-up period and without death in the first 3 days of hospitalization were categorized into the no organ dysfunction group, which represents COVID-19 patients with mild to moderate illness severity.

### Exposure

The study examined the treatment strategy of using antivirals versus not using antivirals. Specifically, there were two primary anti-exposure interests: (1) nirmatrelvir-ritonavir and (2) molnupiravir. Each antiviral group was compared with a control group consisting of patients who did not receive any antiviral treatment during their hospital stay. We used a new-user design, including only patients who had received antiviral treatment within the 3 days of admission [20], and excluded those who began antiviral treatment before admission. Controls were defined as patients who did not use antivirals at any point during the study period. To ensure the selection of new users, we included only patients who had not received antivirals within 7 days prior to admission and excluded those concurrently using both antivirals.

### Follow-Up Period and Outcomes

Patients were followed until one of the following events occurred: death, discharge, or 28 days after admission. The primary outcome of interest was in-hospital mortality and length of stay, defined by the time difference between the date of admission and the end of follow-up.

### Covariates

Baseline covariates included: age, sex, residency in care homes for older people, health status over the past 2 years as indicated by the Charlson Comorbidity Index, prior antibiotic use, the calendar year and month of COVID-19 diagnosis, and socioeconomic status as indicated by the Social Deprivation Index (SDI) of their residential address [21,22]. An SDI quartile of Q4 indicated the most deprived, while Q1 indicated the least deprived. To account for evolving COVID-19 characteristics due to emerging variants, the month of admission was included in the propensity score model. Patients with missing data were excluded from analyses.

### Statistical Analysis

We hypothesized that antiviral use (nirmatrelvir-ritonavir or molnupiravir) would be associated with a decreased risk of mortality and a shorter length of stay compared with no antiviral use. Propensity score matching was used to balance baseline characteristics among the groups, using a 1:2 ratio with the nearest neighbor algorithm. A caliper width of 0.2 was chosen to optimize matching [23]. Covariate balance postmatching was evaluated using standardized mean differences (SMD), with a SMD value <0.2 considered indicative of balance. Descriptive statistics were reported as mean (SD) and count (percentage [%]) wherever appropriate. Estimates for in-hospital mortality were presented as hazard ratios (HR) with 95% CIs, derived from a univariate Cox proportional hazards model. The proportional hazards assumption was tested using the Schoenfeld Residual test, which confirmed that the assumption was met. Length of stay estimates were expressed as mean difference with 95% CI. Absolute risk difference, reported as percentages (%) with 95% CI, is calculated as the difference between absolute risks of antiviral and control groups, which are individually calculated as (number of events / number of patients × 100%).

Additionally, 2 sensitivity analyses were conducted: excluding patients who received adjuvant therapies such as remdesivir, interferon-beta 1b, tocilizumab, baricitinib, and plasma infusions during hospitalization; and a landmark analysis of in-hospital mortality on the 3rd day of admission.

All analyses were performed using R (version 4.4.1; R Foundation for Statistical Computing). All tests were 2-tailed, and a *P* value <.05 was considered statistically significant. Generative artificial intelligence was not used in any portion of the composition of the manuscript.

### Ethical Considerations

This study received approval from the Hospital Authority Hong Kong West Cluster/The University of Hong Kong Institutional Review Board (UW 20‐112) and was carried out in accordance with the Helsinki Declaration. The requirement for obtaining informed consent from patients was waived by the institutional review board, as all patient data collection and extraction was anonymized. No compensation was received for human participants’ research, and no identification of individual participants is possible in the study materials.

## Results

### Study Population

Following the exclusion of patients who did not meet the culture-negative criteria or fit within defined organ dysfunction groups, our analysis encompassed 15,599 patients (Table 1). The mean age of the cohort was 75.10 (SD 15.86) years. Among those identified with organ dysfunction, 511 patients experienced circulatory shock, 5236 had respiratory failure, 1053 had acute kidney injury, 880 had coagulopathy, and 162 exhibited acute liver impairment. Notably, respiratory failure frequently co-occurred with other organ dysfunctions, manifesting in 386 patients (76.1%) with circulatory shock, 632 (60.5%) patients with acute kidney injury, 521 (59.5%) patients with coagulopathy, and 76 (46.9%) patients with acute liver impairment. A total of 5695 patients had at least one type of organ dysfunction upon admission, classifying them under the composite organ dysfunction group.

**Table 1.** Baseline characteristics of COVID-19 patients before propensity score matching.^a^

	Circulatory shock (n=511)	Respiratory failure (n=5236)	Acute kidney injury (n=1053)	Coagulopathy (n=880)	Acute liver impairment (n=162)	No organ dysfunction (n=7757)	Composite organ dysfunction (n=5695)
Sex (male), n (%)	351 (68.7)	3117 (59.5)	638 (60.6)	528 (60.0)	112 (69.1)	3930 (50.7)	3384 (59.4)
Age (years), mean (SD)	73.22 (13.83)	79.14 (13.08)	76.73 (13.96)	76.58 (13.66)	72.96 (16.09)	72.16 (17.44)	78.28 (13.50)
Admission of aged care resident, n (**%**)	60 (11.7)	1189 (22.7)	164 (15.6)	118 (13.4)	14 (8.6)	1280 (16.5)	1224 (21.5)
Charlson Comorbidity Index, n (**%**)							
0	53 (10.4)	132 (2.5)	34 (3.2)	31 (3.5)	12 (7.4)	805 (10.4)	166 (2.9)
1	30 (5.9)	187 (3.6)	50 (4.7)	30 (3.4)	8 (4.9)	553 (7.1)	232 (4.1)
2	93 (18.2)	522 (10.0)	140 (13.3)	113 (12.8)	23 (14.2)	1058 (13.6)	621 (10.9)
3	144 (28.2)	950 (18.1)	208 (19.8)	188 (21.4)	41 (25.3)	1551 (20.0)	1073 (18.8)
4+	222 (43.4)	3445 (65.8)	621 (59.0)	518 (58.9)	78 (48.1)	3790 (48.9)	3603 (63.3)
Social Deprivation Index, n (**%**)							
Q1^b^	22 (4.3)	754 (14.4)	121 (11.5)	96 (10.9)	26 (16.0)	1020 (13.1)	779 (13.7)
Q2	30 (5.9)	1795 (34.3)	395 (37.5)	325 (36.9)	58 (35.8)	2794 (36.0)	2011 (35.3)
Q3	93 (18.2)	1899 (36.3)	408 (38.7)	342 (38.9)	56 (34.6)	2853 (36.8)	2068 (36.3)
Q4	222 (43.4)	788 (15.0)	129 (12.3)	117 (13.3)	22 (13.6)	1090 (14.1)	837 (14.7)
Previous antibiotic use, n (%)	341 (66.7)	3413 (65.2)	603 (57.3)	497 (56.5)	87 (53.7)	3480 (44.9)	3540 (62.2)
Antiviral use upon admission, n (%)							
Nirmatrelvir-ritonavir	37 (7.2)	490 (9.4)	92 (8.7)	88 (10.0)	19 (11.7)	2487 (32.1)	596 (10.5)
Molnupiravir	35 (6.8)	329 (6.3)	123 (11.7)	109 (12.4)	23 (14.2)	1596 (20.6)	482 (8.5)
Coexisting organ dysfunction upon admission, n (%)							
Circulatory shock	—^c^	386 (7.4)	189 (18.1)	116 (13.3)	18 (11.1)	—	—
Respiratory failure	386 (76.1)	—	632 (60.5)	521 (59.5)	76 (46.9)	—	—
Acute kidney injury	189 (37.0)	632 (12.1)	—	344 (39.1)	32 (19.8)	—	—
Coagulopathy	116 (22.7)	521 (10.0)	344 (32.8)	—	37 (22.8)	—	—
Acute liver impairment	18 (3.5)	76 (1.5)	32 (3.1)	37 (4.2)	—	—	—

a COVID-19 patients were retrospectively identified from electronic health care records in Hong Kong. The most common organ dysfunction presenting in patients was respiratory failure (n=5236), followed by acute kidney injury (n=1053) and coagulopathy (n=880). Most patients had used antibiotics before receiving antiviral treatment.

b Q: Quartile.

c Not applicable.

### Propensity Score Matching

To ensure comparability between treatment groups, propensity score matching was conducted, resulting in 14 well-balanced cohorts, with all matched variables achieving an SMD of less than 0.2. The distribution of propensity score postmatching is depicted in Figure S2 in Multimedia Appendix 1.

### In-Hospital Mortality

In-hospital mortality data, along with cohort sizes and death counts across comparator groups, are detailed in Table 2. Antiviral use was associated with significantly decreased mortality in patients without organ dysfunction, for both nirmatrelvir-ritonavir (HR 0.17, 95% CI 0.05-0.56) and molnupiravir (HR 0.29, 95% CI 0.15-0.56). Molnupiravir was associated with a significantly reduced mortality hazard (HR 0.75, 95% CI 0.58-0.96) among patients with composite organ dysfunction, and a marginally significant reduction (HR 0.69, 95% CI 0.46-1.02) among patients with respiratory failures. Nirmatrelvir-ritonavir was also associated with reduced absolute mortality risk among patients with respiratory failure (absolute risk difference: 9.5%, 95% CI 6.26%-12.72%) and composite organ dysfunction (absolute risk difference: 6.0%, 95% CI 3.18%-8.91%). In other subgroups of patients with individual organ dysfunction, the reductions in mortality were not statistically significant and had wide CIs.

**Table 2.** In-hospital mortality grouped by organ dysfunction and use of antiviral medication after propensity score matching.

Treatment	Antiviral				Control					
	Patients, n	Events, n	Patient-days, n	Absolute risk, %	Patients, n	Events, n	Patient-days, n	Absolute risk, %	Absolute risk difference, % (95% CI)	Hazard ratio (95% CI)
Nirmatrelvir-ritonavir										
Circulatory shock	34	5	501	14.7	66	18	1120	27.3	12.6 (−3.47 to 28.60)	0.67 (0.25 to 1.83)
Respiratory failure	490	34	3789	6.9	980	161	10,879	16.4	9.5 (6.26 to 12.72)^a^	0.73 (0.51 to 1.06)
Acute kidney injury	87	17	1468	19.5	172	52	4149	30.2	10.7 (−0.10 to 21.48)	0.77 (0.44 to 1.34)
Coagulopathy	88	13	1570	14.8	173	33	4304	19.1	4.3 (−5.14 to 13.75)	0.98 (0.52 to 1.88)
Acute liver impairment	14	0	148	0.0	26	3	409	11.5	11.5 (−0.74 to 23.82)	—^b^
No organ dysfunction	1581	3	6957	0.2	1661	47	13,152	2.8	2.6 (1.81 to 3.47)^a^	0.17 (0.05 to 0.56)^a^
Composite organ dysfunction	725	71	7637	9.8	1446	229	20,966	15.8	6.0 (3.18 to 8.91)^a^	0.88 (0.67 to 1.14)
Molnupiravir										
Circulatory shock	34	7	619	20.6	65	11	958	16.9	−3.69 (−20.03 to 12.70)	1.21 (0.47 to 3.13)
Respiratory failure	327	33	3657	10.1	653	97	7226	14.9	4.8 (0.51 to 9.02)	0.69 (0.46 to 1.02)
Acute kidney injury	123	20	2360	16.3	242	73	6273	30.2	13.9 (5.19 to 22.62)^a^	0.77 (0.44 to 1.34)
Coagulopathy	109	18	2110	16.5	213	50	5333	23.5	7.0 (−2.04 to 15.96)	0.90 (0.53 to 1.55)
Acute liver impairment	23	4	254	17.4	43	7	698	16.3	−1.09 (−20.13 to 17.91)	0.70 (0.43 to 1.15)
No organ dysfunction	1533	10	8927	0.7	1801	64	14,568	3.59	2.9 (1.96 to 3.85)^a^	0.29 (0.15 to 0.56)^a^
Composite organ dysfunction	619	83	9036	13.4	1237	225	18,641	18.2	4.8 (1.34 to 8.22)^a^	0.75 (0.58 to 0.96)^a^

a Denotes statistically significant. Absolute risk difference is calculated as the difference between absolute risks of antiviral and control groups, which are individually calculated as (number of events / number of patients×100%). Reduced in-hospital mortality was seen for nirmatrelvir-ritonavir users in respiratory failure, composite organ dysfunction, and no organ dysfunction; and for molnupiravir users in acute kidney injury, composite organ dysfunction and no organ dysfunction. Due to small sample size in the acute liver impairment group using nirmatrelvir-ritonavir, hazard ratio was not calculated for that group.

b Not available.

### Length of Stay

Our analysis demonstrated that the use of both nirmatrelvir-ritonavir and molnupiravir was associated with a reduced length of hospital stay for patients with various types of organ dysfunction at admission, detailed in Table 3. Specifically, nirmatrelvir-ritonavir significantly reduced the length of stay in patients with respiratory failure by an average (mean) of 3.37 (95% CI 2.32-4.42) days; acute kidney injury by 7.25 (95% CI 2.97-11.52) days; coagulopathy by 7.04 (95% CI 2.44-11.64) days, and in those without organ dysfunction by 3.52 (95% CI 2.99-4.05) days. Additionally, it reduced the length of stay by 3.97 days in patients with composite organ dysfunction (95% CI 2.77-5.17). However, no significant reduction was observed in patients with circulatory shock (2.23, 95% CI −4.54 to 9.00 days) and acute liver impairment (5.16, 95% CI −0.96 to 11.28 days).

**Table 3.** Length of stay (days) grouped by organ dysfunction and use of antiviral medication.

Treatment	Antiviral, mean (SD)	Control, mean (SD)	Mean difference (95% CI)
Nirmatrelvir-ritonavir			
Circulatory shock	14.74 (16.26)	16.97 (15.66)	2.23 (−4.54 to 9.00)
Respiratory failure	7.73 (8.62)	11.1 (11.46)	3.37 (2.32 to 4.42)^a^
Acute kidney injury	16.87 (12.58)	24.12 (22.3)	7.25 (2.97 to 11.52)^a^
Coagulopathy	17.84 (13.7)	24.88 (23.96)	7.04 (2.44 to 11.64)^a^
Acute liver impairment	10.57 (8.36)	15.73 (10.22)	5.16 (−0.96 to 11.28)
No organ dysfunction	4.40 (4.17)	7.92 (10.18)	3.52 (2.99 to 4.05)^a^
Composite organ dysfunction	10.5 (11.2)	14.5 (17.0)	3.97 (2.77 to 5.17)^a^
Molnupiravir			
Circulatory shock	18.21 (22.53)	14.74 (14.86)	−3.47 (−12.07 to 5.14)
Respiratory failure	11.18 (11.26)	11.07 (10.8)	−0.12 (−1.60 to 1.36)
Acute kidney injury	19.19 (17.53)	25.92 (24.03)	6.73 (2.39 to 11.08)^a^
Coagulopathy	19.36 (16.94)	25.04 (23.34)	5.68 (1.20 to 10.16)^a^
Acute liver impairment	11.04 (7.85)	16.23 (13.82)	5.19 (−0.14 to 10.52)
No organ dysfunction	5.82 (5.93)	8.09 (9.99)	2.27 (1.72 to 2.81)^a^
Composite organ dysfunction	14.6 (14.9)	15.1 (17.9)	0.47 (−1.06 to 2.01)

a Denotes statistically significant. Nirmatrelvir-ritonavir reduced length of stay in respiratory failure, acute kidney injury; while molnupiravir reduced length of stay in acute kidney injury and coagulopathy.

Molnupiravir also showed a significant reduction in the length of stay for patients with acute kidney injury by an average (mean) of 6.7 (95% CI 2.39-11.08) days, coagulopathy by 5.68 (95% CI 1.20-10.16) days, and in those without organ dysfunction by 2.27 (95% CI 1.72-2.81) days. However, no significant reduction was observed for circulatory shock (−3.47, 95% CI −12.07 to 5.14 days), respiratory failure (−0.12, 95% CI −1.60 to 1.36 days), acute liver impairment (5.19, 95% CI −0.14 to 10.52 days), or composite organ dysfunction (0.47, 95% CI −1.06 to 2.01 days).

### Sensitivity Analysis

Results excluding patients who received adjuvant therapy are described in Tables S3 and S4 in Multimedia Appendix 1.

The sensitivity analysis revealed reduced mortality associated with nirmatrelvir-ritonavir in respiratory failure (absolute risk difference: 8.3%, 95% CI 2.4%-14.2%) and molnupiravir use in patients with coagulopathy (absolute risk difference: 13.0%, 95% CI 2.2%-23.8%). Consistent with the main analysis, both antivirals were associated with decreased mortality in patients without organ dysfunction and composite organ dysfunction. 

Nirmatrelvir-ritonavir use was associated with a decreased length of stay in patients with respiratory failure by an average of 8.4 (95% CI 5.1-11.7) days, acute kidney injury by 11.6 (95% CI 4.1-19.1) days, coagulopathy by 12.8 (95% CI 4.7-20.9) days, and those without organ dysfunction by 3.7 (95% CI 3.1-4.3) days. Molnupiravir was associated with a decreased length of stay in patients with acute kidney injury by an average of 8.1 (95% CI 1.5-14.7) days, coagulopathy by 7.8 (95% CI 0.2-15.4) days, and those without organ dysfunction by 2.2 (95% CI 0.5-6.2) days. Nirmatrelvir-ritonavir was associated with decreased length of stay in patients with composite organ dysfunction by an average of 9.8 (95% CI 7.3-12.2) days, while molnupiravir was associated with an average reduction of 3.4 (95% CI, 0.5-6.2) days in the length of stay among patients with composite organ dysfunction.

Results of the landmark analysis 3 days after hospitalization are available in Table S5 in Multimedia Appendix 1. Consistent with the main analysis, nirmatrelvir-ritonavir was associated with decreased mortality in respiratory failure (HR 0.43, 95% CI 0.22-0.82) and composite organ dysfunction (HR 0.42, 95% CI 0.22-0.80), as well as lower risk of circulatory shock (absolute risk difference: 13.7%, 95% CI 3.1-24.4). Molnupiravir was similarly associated with decreased mortality in respiratory failure (HR 0.19, 95% CI 0.08-0.47) and composite organ dysfunction (HR 0.19, 95% CI 0.08-0.48).

## Discussion

### Principal Findings

In our study of patients diagnosed with culture-negative viral sepsis, antiviral use was associated with reduced mortality among those without any organ dysfunction, with respiratory failure, or with composite organ dysfunction. The strongest reduction in mortality was observed among patients without any organ dysfunction. The use of these antivirals was associated with reduced length of stay across most types of organ dysfunction, except for circulatory shock and acute liver impairment. Importantly, the decreased length of stay was consistent regardless of whether patients received adjuvant therapies including remdesivir, interferon-beta 1b, tocilizumab, baricitinib, or plasma infusions. These findings build on our recent research using an inverse probability weighting approach to evaluate the impact of antivirals in preventing the onset of new organ dysfunction [24].

Our results are in line with previous studies that have investigated the utility of treating COVID-19-associated organ dysfunction. For patients hospitalized with COVID-19, both nirmatrelvir-ritonavir and molnupiravir have been associated with a decreased risk of developing new organ dysfunctions, such as circulatory shock and respiratory failure [24]. Additionally, earlier research has shown that antivirals can reduce the length of hospital stay and accelerate viral clearance times [17,25,26].

The 2021 Surviving Sepsis Campaign COVID-19 guideline did not provide specific recommendations for the newly introduced nirmatrelvir-ritonavir and molnupiravir [14]. They did recommend intravenous remdesivir due to its ability to reduce hospital stay and shorten time to clinical recovery, although it did not reduce mortality benefit across different disease severity strata [27]. In our study, the antivirals nirmatrelvir-ritonavir and molnupiravir showed a mortality benefit for those with mild to moderate disease, with stronger effects in patients without presenting organ dysfunction.

The Infectious Diseases Society of America Guidelines on the Treatment and Management of Patients with COVID-19 advocate for the timely administration of antivirals, including nirmatrelvir-ritonavir and molnupiravir, within 5 to 7 days of symptom onset [15]. This period is deemed most effective due to high viral load and immature immune response characteristic of early infection. These antivirals are specifically recommended for patients with mild to moderate COVID-19 [28,29]. However, many patients are admitted with pre-existing organ dysfunction, indicative of an excessive inflammatory response typical of moderate to severe disease, where the efficacy of antivirals diminishes. Our findings highlight that organ dysfunction constitutes a significant proportion of COVID-19 inpatient deaths. While anti-inflammatory treatments, such as corticosteroids or tocilizumab, are recommended for critically ill COVID-19 patients [14], our data suggest that antivirals still have clinical utility by reducing the length of hospital stay and hastening recovery.

For patients with suspected sepsis but unconfirmed infection, it is recommended to consider alternative diagnoses and the discontinuation of antibiotics [14]. Given the high prevalence of COVID-19, particularly among older adults, it is crucial to consider COVID-19 as a potential source of sepsis when bacterial, parasitic, or fungal causes of infection are not established.

This study has several limitations. First, the study did not account for the varying severity of organ dysfunction due to the heterogeneous clinical presentation of sepsis in undifferentiated COVID-19 patients. It is plausible that antivirals could demonstrate a mortality benefit in patients with mild organ dysfunction. Second, the study assumed that all patients initiated on antivirals completed the full course of treatment. Thirdly, this study excluded patients who did not have blood culture records and those with positive blood culture records; thus, the results may not be generalizable to all COVID-19 inpatients. Last, COVID-19 diagnoses were identified from *ICD-9* codes. Nevertheless, CDARS has previously demonstrated high coding accuracy and data completeness in prior studies [30,31].

### Conclusion

Using electronic health data representative of Hong Kong residents, we investigated the effect of nirmatrelvir-ritonavir and molnupiravir on reducing mortality and hospital stay among patients with or without early-onset organ failures. Their association with reduced mortality and hospital stay underscores their potential role in expediting clinical recovery.

## Supplementary material

10.2196/72124 Multimedia Appendix 1 Supplementary publication material.

## Abbreviations

CDARS: Clinical Data Analysis and Reporting System
HR: hazard ratio
*ICD-9-CM*: *International Classification of Diseases, Ninth Revision, Clinical Modification*
SDI: Social Deprivation Index
SMD: standardized mean difference
