# Supplementary material for: Effectiveness of Antivirals Nirmatrelvir-Ritonavir and Molnupiravir in Viral Sepsis: Retrospective Cohort Study
Source: JMIR Public Health Surveill. 2025 Sep 18;11:e72124. doi: 10.2196/72124 (PMC12445620; doi:10.2196/72124)
Supplement: Multimedia Appendix 1 [file publichealth-v11-e72124-s001.docx]

**Table S1: Organisms identified from blood culture not considered as secondary infection**

| Bacillus cereus  Bacillus cereus group  Clostridium perfringens  Coagulase negative Staphylococci  Coagulase negative Staphylococcus  Coagulase -ve staphylococci  Diphtheroid bacilli  Staphylococcus capitis  Staphylococcus caprae  Staphylococcus epidermidis |
| --- |

**Table S2: Organ dysfunction definitions**

Only the laboratory parameters within the timeframe of the first 3 days of hospitalization were considered in the categorization of patients into each organ dysfunction group.

| **Category** | **Definition** |
| --- | --- |
| Circulatory shock | Initiation of a vasopressor or inotrope |
| Respiratory failure | Initiation of invasive mechanical ventilation (identified by ICD-9 procedure codes 93.90, 93.91, 96.70, 96.71, 96.72) OR initiation of dexamethasone |
| Acute kidney injury | Doubling of serum creatinine from the lowest value during hospitalization OR decrease by ≥50% of estimated glomerular filtration rate relative to highest value during hospitalization. |
| Coagulopathy | Platelet count <100 cells/µL AND > 50% decline from highest value during hospitalization. |
| Acute liver impairment | Total bilirubin ≥2.0mg/dL AND increase by 100% from lowest value during hospitalization. |
| No organ dysfunction | Absence of any one of the above secondary outcomes any time during hospitalization and survived first three days of hospitalization |
| Composite organ dysfunction | Presence of any organ dysfunction |

**Figure S1: Cohort selection flow chart**


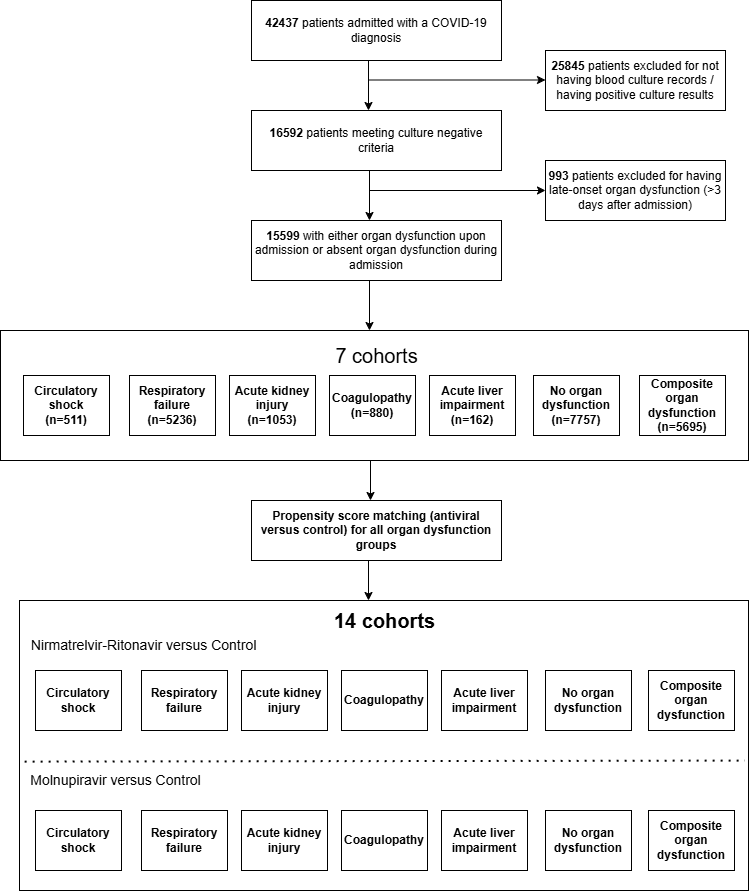


**
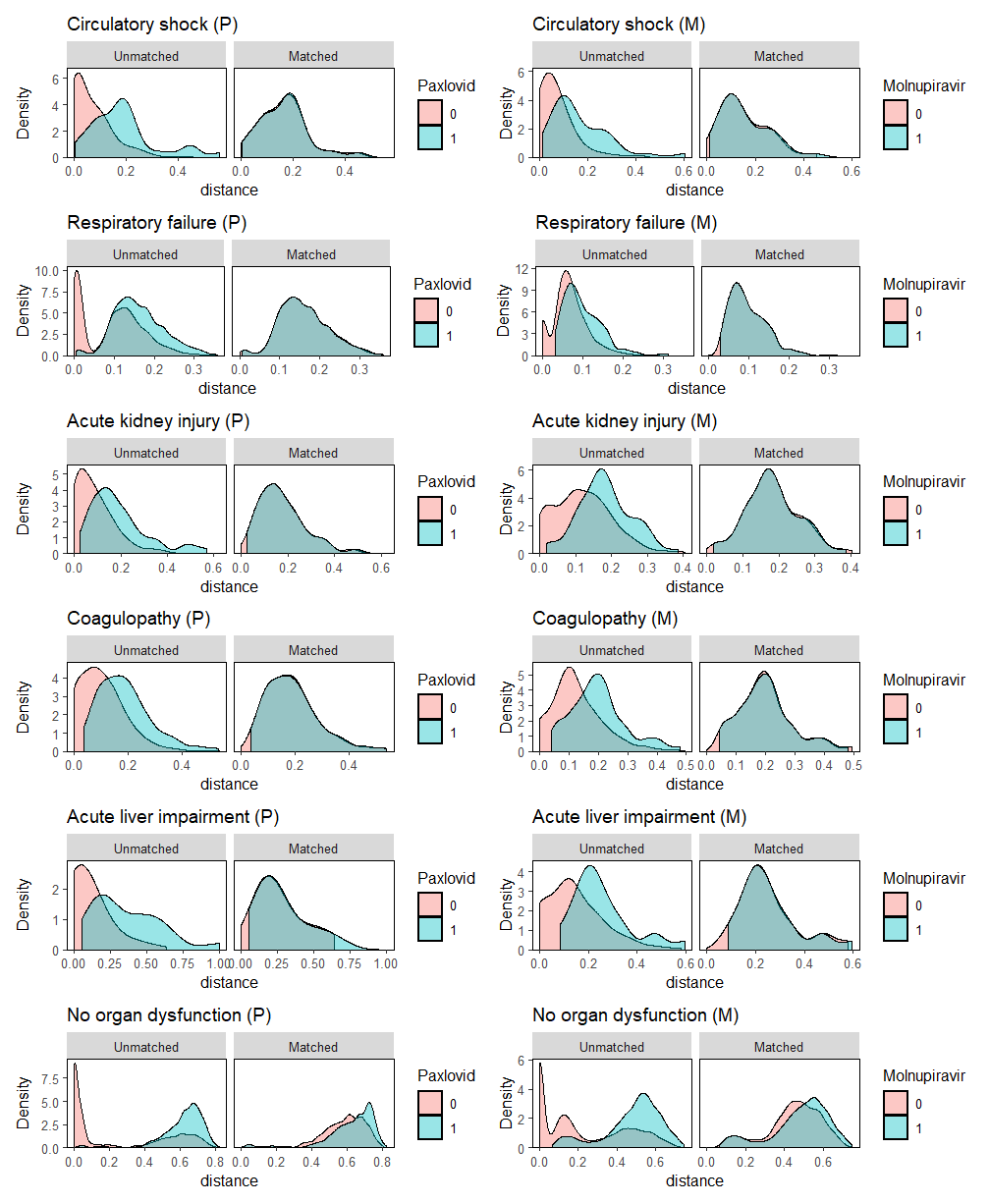
Figure S2a: Distribution balance of propensity scores of organ dysfunction cohorts before and after propensity score matching**

*P, Nirmatrelvir-Ritonavir (Paxlovid); M, Molnupiravir.

**Table S2b: Distribution balance of propensity scores of organ dysfunction cohorts before and after propensity score matching**

**
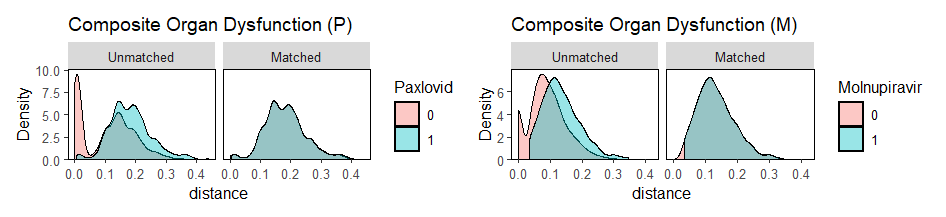
**

*P, Nirmatrelvir-Ritonavir (Paxlovid); M, Molnupiravir.

**Table S3: In-hospital mortality grouped by organ dysfunction and use of antiviral medication after propensity score matching, excluding patients with adjuvant therapy**

|  | **Antiviral** | | | | **Control** | | | |  |  |
| --- | --- | --- | --- | --- | --- | --- | --- | --- | --- | --- |
| **Treatment** | **No. of patients** | **No. of events** | **No. of patient-days** | **Absolute risk (%)** | **No. of patients** | **No. of events** | **No. of patient-days** | **Absolute risk (%)** | **Absolute risk difference (%, 95% CI)** | **Hazard ratio (95% CI)** |
| **Nirmatrelvir-ritonavir** | | | | | | | | | | |
| Circulatory shock | 5 | 1 | 36 | 20.0 | 7 | 0 | 101 | 0.0 | -20.0 (-55.1 to 15.1) | NA |
| Respiratory failure | 126 | 5 | 683 | 4.0 | 179 | 22 | 2476 | 12.3 | 8.3 (2.4 to 14.2)* | 0.78 (0.29 to 2.10) |
| Acute kidney injury | 40 | 5 | 662 | 12.5 | 74 | 7 | 2084 | 9.5 | -3.0 (-15.3 to 9.2) | 1.37 (0.43 to 4.33) |
| Coagulopathy | 42 | 5 | 704 | 11.8 | 61 | 6 | 1805 | 9.8 | -2.1 (-14.4 to 10.3） | 1.87 (0.56 to 6.20) |
| Acute liver impairment | 4 | 0 | 39 | 0.0 | 5 | 1 | 87 | 20.0 | 20.0 (-15.1 to 55.1) | NA |
| No organ dysfunction | 1340 | 1 | 5669 | 0.1 | 1419 | 32 | 11268 | 2.3 | 2.2 (1.4 to 3.0)* | 0.11 (0.01 to 0.80)* |
| Composite organ dysfunction | 235 | 9 | 2033 | 3.8 | 350 | 33 | 6449 | 13.2 | 9.4 (4.5 to 14.2)* | 0.85 (0.40 to 1.78) |
| **Molnupiravir** | | | | | | | | | | |
| Circulatory shock | 5 | 1 | 68 | 20.0 | 8 | 0 | 92 | 0.0 | -20.0 (-55.1 to 15.1) | NA |
| Respiratory failure | 104 | 8 | 1089 | 7.7 | 181 | 18 | 1969 | 9.9 | 2.3 (-4.5 to 9.0) | 0.73 (0.32 to 1.68) |
| Acute kidney injury | 76 | 5 | 1493 | 6.6 | 124 | 18 | 3439 | 14.5 | 7.9 (-0.4 to 16.3) | 0.59 (0.22 to 1.61) |
| Coagulopathy | 57 | 4 | 1164 | 7.0 | 85 | 17 | 2399 | 20.0 | 13.0 (2.2 to 23.8)* | 0.44 (0.15 to 1.31) |
| Acute liver impairment | 5 | 0 | 46 | 0.0 | 7 | 0 | 75 | 0.0 | 0.0 (0.0 to 0.0) | NA |
| No organ dysfunction | 1310 | 6 | 7555 | 0.5 | 1544 | 61 | 12350 | 4.0 | 3.5 (2.5 to 4.5)* | 0.20 (0.08 to 0.46)* |
| Composite organ dysfunction | 236 | 16 | 3313 | 6.8 | 409 | 47 | 7116 | 11.5 | 4.7 (0.3 to 9.2) | 0.68 (0.39 to 1.20) |

*(asterisk) denotes statistically significant. Due to small sample sizes, some hazard ratios were not calculated.

**Table S4: Length of stay (days) grouped by organ dysfunction and use of antiviral medication, excluding patients with adjuvant therapy**

| **Treatment** | **Antiviral**  **Mean (SD)** | **Control**  **Mean (SD)** | **Mean difference (95%CI)** |
| --- | --- | --- | --- |
| **Nirmatrelvir-ritonavir** | | | |
| Circulatory shock | 7.2 (5.3) | 14.4 (14.2) | 7.2 (-6.3 to 20.7) |
| Respiratory failure | 5.4 (6.9) | 13.8 (20.9) | 8.4 (5.1 to 11.7)* |
| Acute kidney injury | 16.6 (9.9) | 28.2 (29.7) | 11.6 (4.1 to 19.1)* |
| Coagulopathy | 16.8 (12.9) | 29.6 (27.8) | 12.8 (4.7 to 20.9)* |
| Acute liver impairment | 9.8 (6.1) | 17.4 (6.0) | 7.7 (-2.1 to 17.4) |
| No organ dysfunction | 4.2 (4.2) | 7.9 (10.3) | 3.7 (3.1 to 4.3)* |
| Composite organ dysfunction | 8.7 (9.1) | 18.4 (19.9) | 9.8 (7.3 to 12.2)* |
| **Molnupiravir** | | | |
| Circulatory shock | 13.6 (21.5) | 11.5 (7.9) | -2.1 (-28.4 to 24.2) |
| Respiratory failure | 10.5 (11.6) | 10.9 (14.8) | 0.4 (-2.7 to 3.5) |
| Acute kidney injury | 19.6 (19.9) | 27.7 (27.1) | 8.1 (1.5 to 14.7)* |
| Coagulopathy | 20.4 (18.9) | 28.2 (27.1) | 7.8 (0.2 to 15.4)* |
| Acute liver impairment | 9.2 (3.8) | 10.7 (5.3) | 1.5 (-4.3 to 7.3) |
| No organ dysfunction | 5.8 (6.1) | 8.0 (9.6) | 2.2 (1.7 to 2.8)* |
| Composite organ dysfunction | 14.0 (15.8) | 17.4 (20.4) | 3.4 (0.5 to 6.2) |

*(asterisk) denotes statistically significant. CI, confidence interval; SD, standard deviation.

**Table S5: Landmark analysis of 3-day In-hospital mortality grouped by organ dysfunction and use of antiviral medication after propensity score matching**

|  | **Antiviral** | | | **Control** | | |  |  |
| --- | --- | --- | --- | --- | --- | --- | --- | --- |
| **Treatment** | **No. of patients** | **No. of events** | **Absolute risk (%)** | **No. of patients** | **No. of events** | **Absolute risk (%)** | **Absolute risk difference (%, 95% CI)** | **Hazard ratio (95% CI)** |
| **Nirmatrelvir-ritonavir** | | | | | | | | |
| Circulatory shock | 34 | 1 | 2.9 | 66 | 11 | 16.7 | 13.70 (3.09 to 24.36)* | 0.17 (0.02 to 1.31) |
| Respiratory failure | 490 | 11 | 2.2 | 980 | 54 | 5.5 | 3.30 (1.33 to 5.21)* | 0.43 (0.22 to 0.82)* |
| Acute kidney injury | 87 | 2 | 2.3 | 172 | 6 | 3.5 | 1.20 (-2.99 to 5.36) | 0.65 (0.13 to 3.21) |
| Coagulopathy | 88 | 0 | 0.0 | 173 | 3 | 1.7 | 1.70 (-0.21 to 3.68) | NA |
| Acute liver impairment | 14 | 0 | 0.0 | 26 | 1 | 3.8 | 3.80 (-3.55 to 11.24) | NA |
| Composite organ dysfunction | 725 | 11 | 1.5 | 1446 | 54 | 3.7 | 2.20 (0.90 to 3.54)* | 0.42 (0.22 to 0.80)* |
| **Molnupiravir** | | | | | | | | |
| Circulatory shock | 34 | 3 | 8.8 | 65 | 14 | 21.5 | 12.7 (-1.09 to 26.53) | 0.38 (0.11 to 1.33) |
| Respiratory failure | 327 | 5 | 1.5 | 653 | 52 | 8.0 | 6.40 (3.97 to 8.90)* | 0.19 (0.08 to 0.47)* |
| Acute kidney injury | 123 | 2 | 1.6 | 242 | 3 | 12 | -0.40 (-3.02 to 2.24) | 1.31 (0.22 to 7.86) |
| Coagulopathy | 109 | 1 | 0.9 | 213 | 2 | 0.9 | 0.00 (-2.18 to 2.23) | 0.97 (0.09 to 10.72) |
| Acute liver impairment | 23 | 0 | 0.0 | 43 | 2 | 4.7 | 4.7 (-1.64 to 10.95) | NA |
| Composite organ dysfunction | 619 | 5 | 0.8 | 1237 | 51 | 4.1 | 3.30 (2.00 to 4.63)* | - 1. (0.08 to 0.48)* |

(*) asterisk denotes statistically significant. The no organ dysfunction group is not included in this analysis as all patients who died within 3 days are excluded.
